# Microbubble-based fabrication of resilient porous ionogels for high-sensitivity pressure sensors

**DOI:** 10.1038/s41378-024-00780-8

**Published:** 2024-11-26

**Authors:** Ziwei Yang, Jingxiao Wang, Xiao Wan, Hongcheng Xu, Chuanyu Zhang, Xiaoke Lu, Weixuan Jing, Chuanfei Guo, Xueyong Wei

**Affiliations:** 1 State Key Laboratory for Manufacturing Systems Engineering, Xi’an Jiaotong University, Xi’an, 710049 China; 2 School of Instrument Science and Technology, Xi’an Jiaotong University, Xi’an, 710049 China; 3 Frontier Institute of Science and Technology, Xi’an Jiaotong University, Xi’an, 710049 China; 4 Department of Materials Science and Engineering, Southern University of Science and Technology, Shenzhen, Guangdong 518055 China

**Keywords:** Electrical and electronic engineering, Materials science

## Abstract

High-sensitivity flexible pressure sensors have obtained extensive attention because of their expanding applications in e-skins and wearable medical devices for various disease diagnoses. As the representative candidate for these sensors, the iontronic microstructure has been widely proven to enhance sensation behaviors such as the sensitivity and limits of detection. However, the fast and tunable fabrication of ionic-porous sensing elastomers remains challenging because of the current template-dissolved or 3D printing methods. Here, we report a microbubble-based fabrication process that enables microporous and resilient-compliance ionogels for high-sensitivity pressure sensors. Periodic motion sliding results in a relative velocity between the imported airflow and the fluid solution, converts the airflow to microbubbles in the high-viscosity ionic fluid and promptly solidifies the fluid into a porous ionogel under ultraviolet exposure. The ultrahigh porosity of up to 95% endows the porous ionogel with superelasticity and a Young’s modulus near 7 kPa. Due to the superelastic compliance and iontronic electrical double-layer effect, the porous ionogel packaged into two electrodes endows the pressure sensor with high sensitivity (684.4 kPa^−1^) over an ultrabroad range (~1 MPa) and a high-pressure resolution of 0.46%. Furthermore, the pressure sensor successfully captures high-yield broad-range signals from the fingertip low-pressure pulses (<1 kPa) to foot high-pressure activities (>500 kPa), even the grasping force of soft machine hands via an array-scanning circuit during object recognition. This microbubble-based fabrication process for porous ionogels paves the way for designing wearable sensors or permeable electronics to monitor and diagnose various diseases.

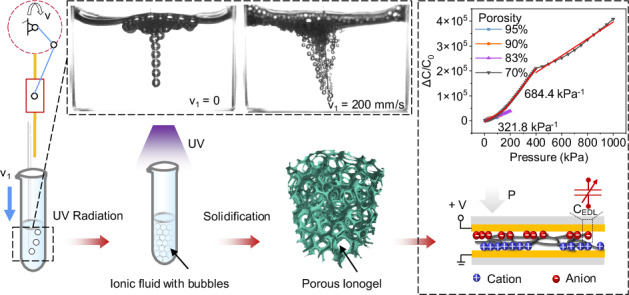

## Introduction

Recently, flexible force sensors that are high-sensitivity, lightweight, and thin have been widely applied in human-machine interfaces^1–3^, health monitoring^4–10^, and wearable electronics^11–20^, where ultralow loads are attached to machines or human skin. Among several force sensors, notable advances have been made in pressure sensors for the trade-off between high sensitivity and working range. To date, iontronic sensing gels, as representative candidates, have been used to increase the sensitivity of high-pressure force sensors^21,22^. When massive ion/charge pairs cluster in parallel around both interfaces between electrodes to form electrical double layers (EDLs), the interfaced ion capacitance can strongly respond to the external pressure. However, there are few flexible sensors that can maintain continuous high sensitivity at large pressures of 1 MPa. Currently, several reports adopt ions embedded into flexible gels to ensure that the sensor continuously responds to large preloads^23–27^. For example, deformation of the fine pillar on the hemisphere of the ionic layer, which is constructed by 3D printing, can compensate for the effect of structural stiffening^28^ and enable a linear response with a sensitivity of 49 kPa^−1^ over a pressure range of up to 485 kPa. These methods, which are based on thin and soft sensing films, are very useful to enhance the sensitivity in a certain force range. However, the continuous elasticity^29^ and compression deformation^30^ of the sensing layer are always limited over a narrow range owing to the hardening of the 2D sensing film lacking wider and continuous mechanical deformation ability, which offers narrow sensitivity-contiguity to the sensor. Therefore, it is crucial to design ionogels for an extended highly sensitive preload range, although often at the expense of high-cost fabrication.

Compared with microstructures (e.g., micropyramidal^31–36^, hemispherical^37^, and pillar^38^) or intrinsic soft materials^39^, porous foam-based elastomers can maintain mechanically continuous high sensitivity and wide compressibility^40^. Commercial foams, such as polyurethane foams, are typically used as dielectric layers after being immersed in an ionic liquid^41,42^. However, the mechanical properties of purchased foams cannot be adjusted, and the ionic liquid may leak from the foam under pressure. Various ordered/unordered micropores have been built via template dissolution^43,44^ or 3D printing^45^ to produce foam skeletons, but many challenges still exist during practical uniform shaping. For example, salt/sugar particles^46^ used as templates can be used to fill resilient polymers or generate pores by evaporation of water through the drying process^47^ to form a random porous geometry, leading to uncontrollable mechanical elasticity, and the 3D printing process has a size effect and material limits; thus, the orderly ionic-pore structure at the μm scale has rarely been reported. Therefore, there is a strong need to develop an effective and controllable fabrication method to prepare programmable iontronic micropores.

Herein, we propose the microbubble-based fabrication of resilient porous ionogels for high-sensitivity pressure sensors over a broadband sensing range. The uniform N_2_ airflow fastened on a periodic-motion slider quickly moves deep into the ionic fluid with high viscosity that retains continuous bubbles from imported gas, which is eventually exposed and solidified in the ultraviolet to form a porous ionogel. The porosity of the ionogel can reach 95%, and the ionogel-based pressure sensors exhibit a maximum sensitivity of 684.4 kPa^−1^ over a wide detection range (~1 MPa) and a high-pressure resolution of 0.46%. The pressure sensor successfully captures high-yield broad-range signals from the fingertip low-pressure pulses (<1 kPa) to foot high-pressure activities ( > 500 kPa), even the grasping force of soft machine hands via an array-scanning circuit during object recognition. This work greatly paves the way for the development of high-performance porous ionogels for soft electronics or human‒machine interfaces.

## Results and discussion

### Design and characterization of the Porous Ionogel

**Fig. 1:**
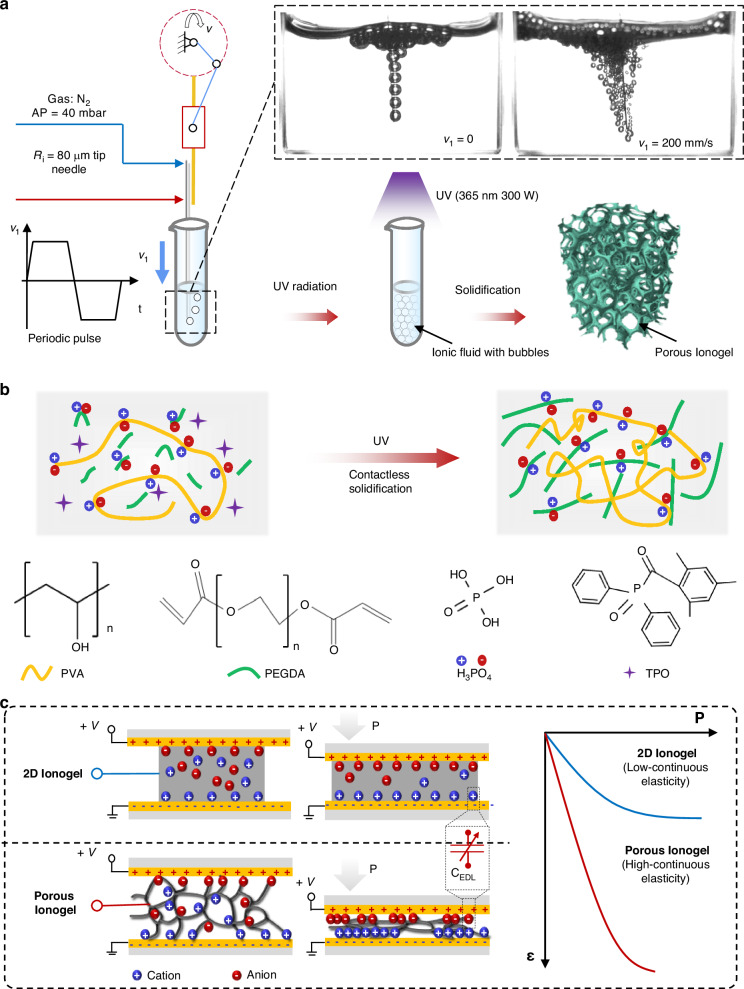
Schematic illustration of the microbubble-based fabrication of ionogels for iontronic sensors. **a** Fabrication of the porous ionogel. **b** Molecular composition of the ionic precursor. **c** Comparison of the sensing mechanism and elasticity of the porous ionogel-based pressure sensor and 2D ionic sensing film-based sensor

The highly resilient-compliance ionogel plays a significant role in force sensors. Porous ionogel with a 3D network skeleton has distinguished advantages compared to traditional microstructures, such as high compressibility^48^, light weight^49^, and absorbing ability^50^. Figure 1a illustrates the fabrication of a porous ionogel. The uniform N_2_ airflow via a needle, which was fastened on a periodic-motion slider, quickly penetrated the ionic fluid with high viscosity and continuous bubbles from the imported gas. The fluid was eventually exposed and solidified in the ultraviolet to form the porous ionogel (see details in “Methods” and Movie S1). For the bubble production, the motion velocity (v_1_) and airflow pressure (AP) are key elements that are related to the bubble size (Fig. S1, Supporting Information). The size decreases with decreasing APs and increasing v_1_ under the uniform ionic fluid and identical depths. Fig. S2 (Supporting Information) shows the experimental setup. When the air pressure is under 40 mbar, the pressure of the solution prevents air from blowing out of the pipe and cannot generate bubbles. Thus, we selected 40 mbar as the minimum air pressure to produce the smallest bubbles without the periodic motion by the slider.

Unlike traditional drying to cure ionic films, which commonly causes geometry variations^51^ or breaks under mechanical peeling^52^, the photo-crosslinker poly(ethylene glycol) diacrylate (PEGDA) mixed with the ionic precursor PVA/H_3_PO_4_ prevents the bubble collapse during the freeze-thaw solidification for the PVA physical crosslinking (Fig. 1b) and contactlessly builds the porous ionogel under continuous UV exposure (see detailed preparation in “Methods”). The composition of the ionic fluid mainly decides the mechanical elasticity and conductivity, which were fully investigated in the characterization and tests. In contrast, due to the EDL effect, the ionic-film-based capacitive pressure sensor exhibits several magnitude orders higher than conventional capacitive sensors. Our porous ionic layer can perform higher continuous compression than flatter 2D films (with or without microstructure) and continuously withstands a wideband pressure. When two electrodes were electrified with an alternative voltage, massive ion pairs near the interface between the ionic film and electrodes formed an ultrahigh unit-area capacitance to respond to the external pressure under compression (Fig. 1c).

**Fig. 2:**
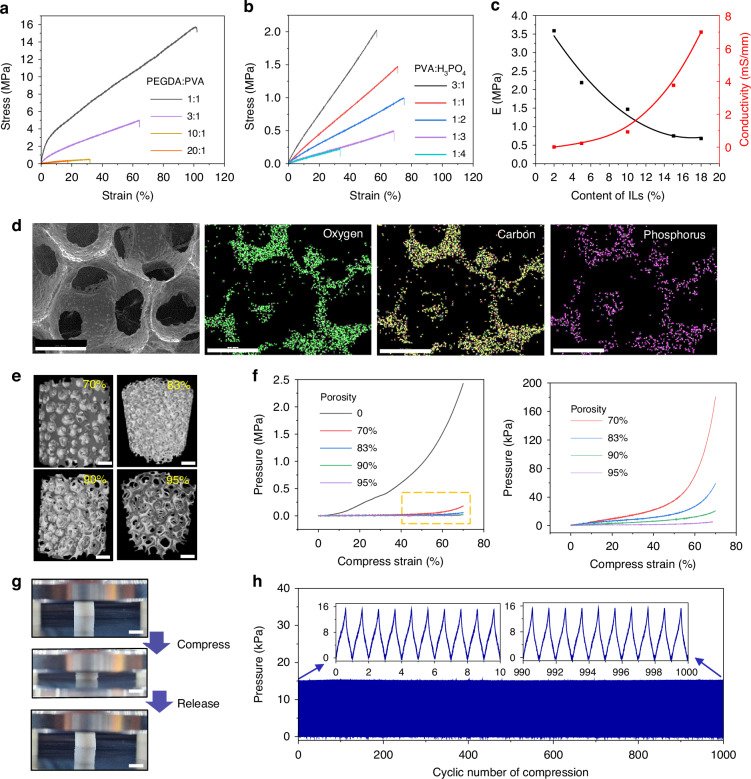
Characterization of the porous ionogel. ε-σ curves with varying weight ratios of (**a**) PEGDA and (**b**) H_3_PO_4_ versus PVA. **c** Young’s modulus and conductivity of the ionogel with various ionic concentrations. **d** Elemental mapping images. Scale bar: 50 μm. **e** 3D reconstructions with different porosities. Scale bar: 300 μm. **f** Compressibility with varying porosity. **g** Optical images of the porous ionogel under 70% compression and release. **h** 1000-cycle durability of the 83% porosity ionogel under a compression pressure of 15 kPa

The composition and geometry of an ionogel affect its mechanical properties, including the elasticity and porosity. The stress–strain curves clearly reveal that the Young’s modulus (slope) decreases with increasing weight ratios of PEGDAs (Fig. 2a), H_3_PO_4_ (Fig. 2b) and decreasing weight ratio of PVAs, which verifies that PVA as the physical skeleton mainly leads to mechanical elasticity. Although the high ion concentration makes intensive conductivity proportional to the EDL capacitance and greatly weakens the mechanical stiffness (Fig. 2c), the high softness of ionogel hardly maintains the robust mechanical rebounding during continuously high loads, so we selected the weight ratios of PEGDA:PVA and H_3_PO_4_:PVA of 3:1 and 2:1 as the optimal composition, respectively. To analyze the effect of the material composition on the electromechanical features, Fig. 2d shows the elemental mapping images of oxygen, carbon, and phosphorus of the porous ionogel. The uniform spectrograms of various elements verify the composition consistency of the porous ionogel, which illustrates its mechanical and electronical stability.

In addition to the material characterization of the ionogel, the mechanical properties are mainly determined by the porous structure^30^. Figure 2e presents 3D reconstructions of porous ionogels with varying porosities obtained from μ-CT scans. Figure 2f shows the compression‒strain curves of the porous ionogel. The compressibility is greatly enhanced by increasing porosity. Notably, the ultrahigh porosity of up to 95% endows the ionogel with superelasticity and a Young’s modulus near 7 kPa, which can make the ionogel far more suitable for various extreme deformations or curved surfaces. Figure 2g presents optical images of the porous ionogel under 70% compression and release. In addition, the 83%-porosity ionogel was compressed 1000 times under a 15-kPa pressure (Fig. 2h), which indicates its mechanical stability. The invariable size of the pore diameter after continuous compression further verifies the superior stability (Fig. S3, Supporting Information). The resistance response during the compression cycles confirms the electrical stability of the porous ionogel, as shown in Fig. S4 (Supporting Information).

### Pressure sensors based on the porous ionogel

**Fig. 3:**
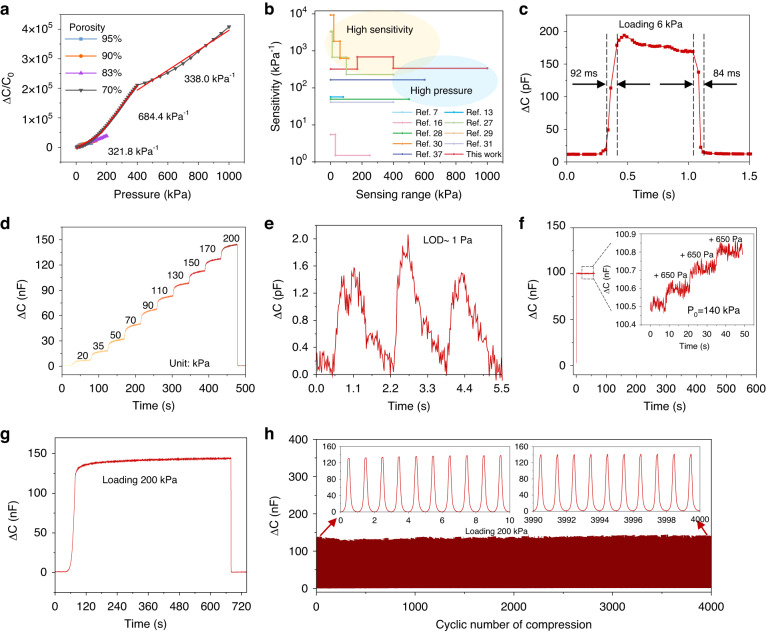
Sensing properties of porous ionogel‑based pressure sensors. **a** Sensitivity of the sensor with four porosities (70%, 83%, 90%, 95%) in different pressure regimes. **b** Comparison of the sensitivity and sensing range of this sensor with those of previous works. **c** Response time at a frequency of 300 kHz. **d** Limit of detection (LOD). **e** Capacitive responses under stepwise increasing pressure from 20 kPa to 200 kPa. **f** Detection of weak pressure (650 Pa) at a large preloaded pressure of 140 kPa. Under a pressure of 200 kPa. **g** Continuous working stability for 10 min and (**h**) long-term durability of the sensor over 4000 cycles

The sensitivity is one of the most critical indicators for pressure sensors and determines the sensing ability to detect the subtle pressure. The sensitivity of a capacitive pressure sensor is defined as S= δ(ΔC/C_0_)/δP, where C_0_ is the initial capacitance without external pressure, ΔC is the capacitive change, and P is the applied pressure. The sensor consists of a typical sandwich-type construction, top/bottom copper electrodes, and an inner ionogel sensing layer. Figure 3a and Fig. S5 show the capacitance-pressure curves of the porous ionogel-based pressure sensor. Importantly, the sensor fully exhibits a high sensitivity higher than 300 kPa^−1^ over the working range near 1 MPa, which is superior to several previous reports (Fig. 3b). When the porosity is near 70%, the sensor exhibits the maximal sensitivity, which requires the applied ionogel to balance the sensing ability and mechanical elasticity. Due to the robust mechanical stability of the ionogel, the four pressure sensors exhibit good consistency, which is important for flexible sensing materials that are prone to creep deformation (Fig. S6, Supporting Information). To observe the response time, a 6-kPa pressure was applied onto the sensor from loading to unloading. The sensor exhibited a fast response time of 92 ms and a recovery time of 84 ms (Fig. 3c), which is almost consistent with the maximum vibration frequency of 5 Hz (Fig. S7, Supporting Information). Furthermore, the pressure sensor can accurately measure a stepwise pressure increment consistent with its varying S-P (Fig. 3d). In addition, the sensor exhibits a low limit of detection (LOD) of approximately 1 Pa (Fig. 3e). Lightweight plastic blocks (weight of 2.5 g, ~650 Pa) were positioned at a preload pressure of 140 kPa, and clear stepped capacitance increments were observed (Fig. 3f). The 0.46% high-pressure resolution indicates excellent force-distinguishing ability. Fig. S8 (Supporting Information) shows the cyclic dynamic bending sensing with the bending radius from 8 mm to 2 mm, which indicates that the capacitance response is sensitive to the bending radius, and the signal is stable after a few bending cycles. During 10 min (Fig. 3g) and 3 h (Fig. S9) of continuous pressure, the output capacitance remained constant. Even under a high pressure of 200 kPa over 4000 cycles, our sensor held long-term durability and a constant value without obvious signal decay (Fig. 3h), both of which verify the superior mechanical robustness.

### Physiological monitoring

**Fig. 4:**
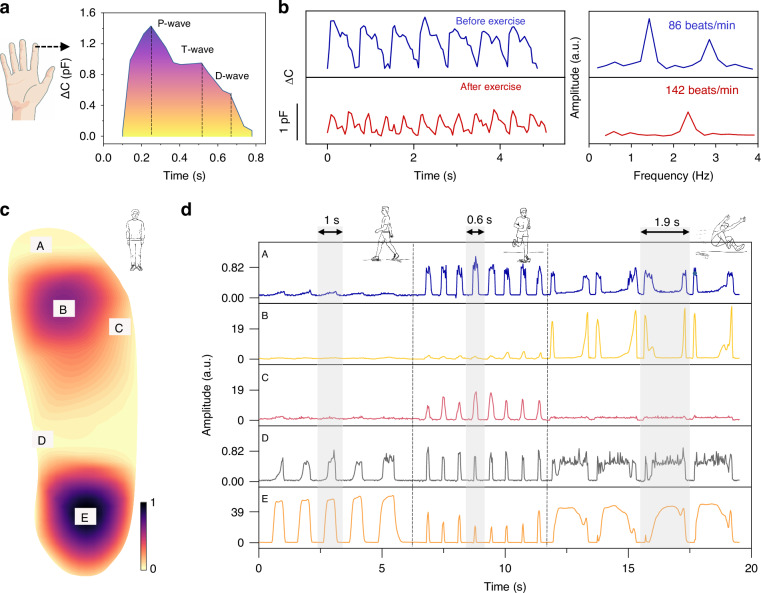
Fingertip pulse and foot pressure monitoring of a healthy female volunteer (45 kg, 28 years old). **a** Pulse wave at the index finger. **b** Continuous pulse wave before and after exercise as the corresponding frequency spectrum by Fourier transformation. **c** Heatmap of the static-standing plantar pressure. **d** Dynamic plantar pressure during walking, running, and leaping

Due to their high sensitivity, broad sensing range, and high resolution, porous-ionogel-based pressure sensors are used to capture vital physiological signals over a broad pressure range from fingertip low-pressure pulses to foot high-pressure activities. As shown in Fig. 4a, our sensor clearly tracks the capacitance fluctuations from weak finger pulses, including P-, T-, and D-waves, as important clinical treatments for cardiovascular diseases. The heartbeat frequency of a healthy female volunteer (28 years old) before and after exercise was 86 and 142 min^−1^, respectively, which is within the standard of a normal person’s pulse rate of 60–150 min^−1^ (Fig. 4b).

In addition, five pressure sensors were attached to the insole and connected with a 5-channel scanning circuit (see the details in “Methods”) to observe the plantar pressure of an adult volunteer (45 kg, 28 years old, female). The heatmap of the plantar pressure (Fig. 4c) shows that the forefoot (B) and hindfoot (E) experienced major weight stress. Our sensor also distinguishes dynamic and continuous pressures in terms of frequency and amplitude during walking, running, and leaping, as shown in Fig. 4d. In particular, the sensor could capture the leaping motion with a quick falling time (<100 ms), which verifies its superior response ability for various fast-force stimuli.

### Spatial force interfacing and object recognition

**Fig. 5:**
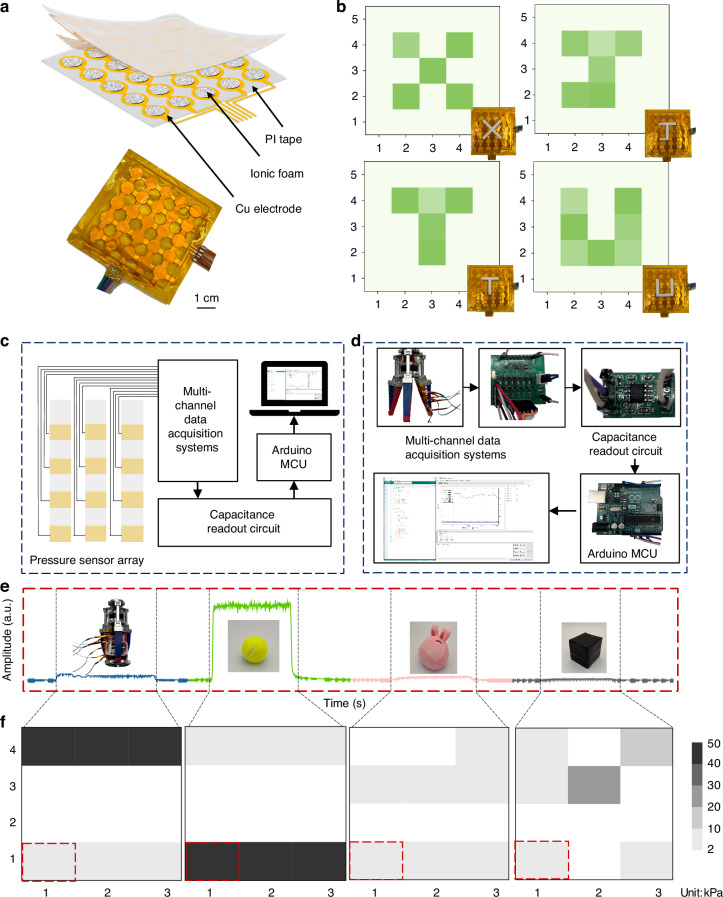
Pressure spatial mapping and object recognition. **a** A designed 5 × 5 sensing array. **b** Pressure map inverted from the capacitance changes as sensors bearing the weight loads of various shapes. **c** Schematic and (**d**) photos of a 3 × 4 array connected with a circuit to obtain signals and detect grasping forces. **e** Digital signal and (**f**) pressure grayscale map of the sensor array while it grasps a cylindrical cup, a spherical tennis ball, an irregular doll, and a stereoscopic box

Figure 5 shows application scenarios of the high sensitivity over the wide working range of the porous ionogel-based sensor. A pressure sensing array with 5 × 5 pixels (Fig. 5a) was built with an infrared femtosecond laser to capture 3D-printed resin loads with various shapes (such as “X”, “J”, “T”, and “U”). Figure 5b shows that the sensing array can simultaneously distinguish the pressure distribution and intensity, which indicates its ability to map forces for practical application.

In addition, a 3 × 4 array was connected with an array scanning circuit to capture the grasping force on the soft three-finger robotic gripper. When the robotic gripper grasped an object with a certain hardness and shape, a dynamic force signal from the machine finger to the attached sensor was transmitted to a multi-channel data acquisition system with periodic-variation capacitance read, a low-power cost MCU, and a digital interface in the PC (Fig. 5c, d). As shown in Fig. 5e, f, our platform can clearly capture the varied hardness and shape of four objects: a cylindrical cup, a spherical tennis ball, an irregular doll, and a stereoscopic box. The grayscale map intuitively depicts the gripping force distribution and can be used to track the dynamic force changes to detect the grasping and releasing actions. The capacitance rapidly increases at the initial contact, and the deformation of the flexible object increases the response time.

## Conclusion

In summary, this work successfully demonstrated the novel microbubble-based fabrication of resilient porous ionogels for high-sensitivity pressure sensors. The ultrahigh porosity of up to 95% endows the ionogel with superelasticity and a Young’s modulus near 7 kPa. The pressure sensor based on the porous ionogel exhibited prominent sensitivity (684.4 kPa^−1^) over an ultrabroad sensing range (~1 MPa) and a high-pressure resolution of 0.46%. These superior behaviors permit wide sensations for vital physiological activities from fingertip low-pressure pulses to foot high-pressure activities, even the grasping force of soft machine hands during object recognition. This work greatly paves the way to develop high-performance porous ionogels for soft electronics and human-machine interfaces.

## Methods

### Preparation of the porous ionogel

First, 0.3 g of polyvinyl alcohol (PVA, Mw=145 000, purchased from Shanghai Aladdin Biochemical Technology Co., Ltd.) and 1 g of poly(ethylene glycol) diacrylate (PEGDA, Mn=700, from Sigma‒Aldrich) were dissolved in 4 g of deionized water at 80 °C for 1 h. Then, 0.6 g of phosphoric acid (H_3_PO_4_, AR, from Shanghai Aladdin Biochemical Technology Co., Ltd.) as an ionic liquid, 0.02 g of diphenyl(2,4,6-trimethylbenzoyl)phosphine oxide (TPO, 97%, from Shanghai Aladdin Biochemical Technology Co., Ltd.) as a photoinitiator, and 0.02 g of sodium dodecyl sulfate (SDS, AR, from Shanghai Aladdin Biochemical Technology Co., Ltd.) as a surfactant were added to the solution and stirred at 80 °C for 1 h. Subsequently, N_2_ airflow was injected into the solution through a customized stainless steel capillary with an 80-μm inner radius, which was connected to a Teflon gas pipe and settled on the slider of a crank slider mechanism. The input gas pressure by the air pump was set at 40 mbar. Movie S1 presents the process of bubble generation when the air pressure was set at 40 mbar and v = 400 rpm. After exposure to 365-nm ultraviolet (UV) light (300 W) for 1 min, a porous iongel formed. For comparison, different weight ratios of PEGDA:PVA (1:1, 3:1, 10:1, and 20:1) and different contents of H_3_PO_4_ (2 wt%, 5 wt%, 10 wt%, 15 wt%, and 18 wt%) were used. By adjusting the revolutions per minute of the crank (100 rpm, 200 rpm, 300 rpm, and 400 rpm), various porosities (70%, 83%, 90%, and 95%) were achieved. Afterwards, the porous gel was dried in a constant-humidity chamber.

### Fabrication of the flexible sensor and sensor array

A circular film of 6 mm in radius and 600 μm in thickness was cut from the porous ionogel. Cu-PI films (50-μm-thick Cu coating layer and 55-μm-thick PI substrate) were cut using a laser cutter (MC-F350A, power of 550 W and a 1000-mm s^−1^ cutting speed for 300 repetitions) into a circular shape of 8 mm in diameter, while the PI film was nondestructive. For the 5 × 5 sensor array, the electrodes were patterned in a 5 × 5 array with a spacing of 3 mm. The wire was bonded on the side of Cu of the Cu-PI film by welding. Afterwards, the porous ionogel was sandwiched between two Cu electrodes and packaged in a PI film.

### Characterization and measurements

Tensile and compression testing was implemented using an electromechanical universal testing machine (ETM 103 A) with a computer-controlled platform. The conductivity was measured using the Kelvin four-terminal sensing method. The modulus of the ionogel was the slope of the first 20% strain of the tensile testing curve. The elemental mapping was performed via energy dispersive spectroscopy (EDS) with a scanning electron microscope (SEM, SU-8010). The microstructures of the porous ionogel were characterized via μ-CT (Y.CHEETAH). The porosity was calculated using the VGSTUDIO MAX software. An LCR meter (E4980AL, KEYSIGHT) was used to measure the capacitance of the pressure sensor.

### Analog circuit design

The capacitance of the sensor in the array was sequentially measured through the gating switch. The circuit used a 555 timer to convert the capacitance into the frequency; then, each signal was processed in the Arduino MCU.

## Supplementary information

Video

Supplementary Information
