# Supplementary material for: Microbubble-based fabrication of resilient porous ionogels for high-sensitivity pressure sensors
Source: Microsyst Nanoeng. 2024 Nov 26;10:177. doi: 10.1038/s41378-024-00780-8 (PMC11589707; doi:10.1038/s41378-024-00780-8)
Supplement: Supplementary file 2 — Supplementary Information [file 41378_2024_780_MOESM2_ESM.docx]

Supporting Information

**Microbubble-Based Fabrication of Resilient Porous Ionogel for High-Sensitivity Pressure Sensor**

*Ziwei Yang, Jingxiao Wang, Xiao Wan, Hongcheng Xu,Chuanyu Zhang, Xiaoke Lu, Weixuan Jing, Chuanfei Guo, and Xueyong Wei **

^1^State Key Laboratory for Manufacturing Systems Engineering, Xi’an Jiaotong University, Xi’an 710049, China

^2^School of Instrument Science and Technology, Xi’an Jiaotong University, Xi’an 710049, China

^3^Frontier Institute of Science and Technology, Xi’an Jiaotong University, Xi’an 710049, China

^4^Department of Materials Science and Engineering, Southern University of Science and Technology, Shenzhen Guangdong 518055, China

^†^Z. Yang and J. Wang contributed equally to this work.

Corresponding author’s Email: [seanwei@mail.xjtu.edu.cn](mailto:seanwei@mail.xjtu.edu.cn)

**Figure S1.** Effect of the experimental parameters. a) Images of bubble size varying with air pressure without movement. b) Images of bubble size varying with the rotation speed of the crank with the air pressure fixed at 40 mbar.

**
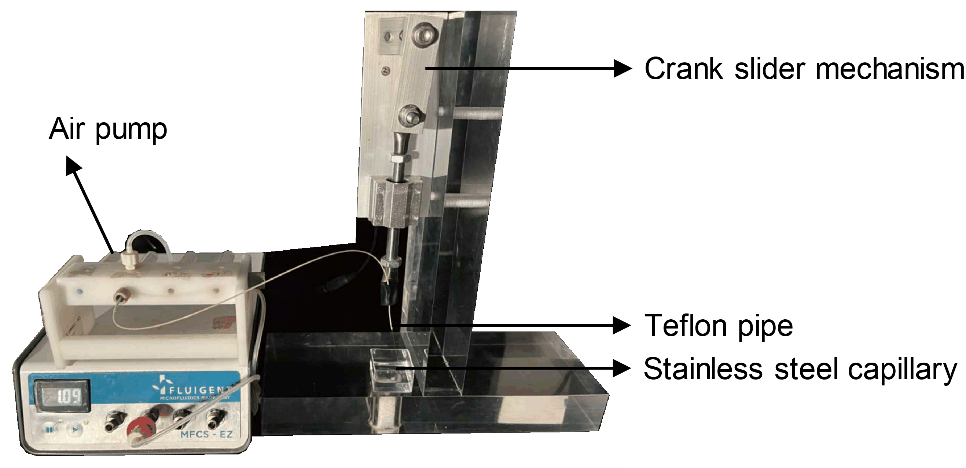
**

**Figure S2.** The photo of the experimental setup for bubble generation in ionic solution.

**Figure S3.** The diameter distribution of the pores before (left) and after (right) the repeated compression.

**Figure S4.** Stability test for electrical properties of porous ionogel.

**Figure S5.** The capacitance-pressure curves of the porous ionogel-based pressure sensor within 200 kPa.

**Figure S6.** The capacitance-pressure curve of four pressure sensors based on the films derived from the foam with the porosity of 70%.

**Figure S7.** Capacitance response at different vibration frequencies.

**Figure S8.** Cyclic dynamic bending sensing with the bending radius from 8 mm to 2 mm

**Figure S9.** Duration of the pressure sensors under a high pressure of 600 kPa for 3 h

**Movie S1.** The process of the bubble generation when the air pressure was set at 40 mbar and the v = 400 rpm. The video playback speed is 3/220 times that of the real experiment.
